# Pseudonymisation of neuroimages and data protection: *Increasing access to data while retaining scientific utility*

**DOI:** 10.1016/j.ynirp.2021.100053

**Published:** 2021-09-15

**Authors:** Damian Eke, Ida E.J. Aasebø, Simisola Akintoye, William Knight, Alexandros Karakasidis, Ezequiel Mikulan, Paschal Ochang, George Ogoh, Robert Oostenveld, Andrea Pigorini, Bernd Carsten Stahl, Tonya White, Lyuba Zehl

**Affiliations:** a Centre for Computing and Social Responsibility, De Montfort University, Leicester, UK; b University of Oslo, Norway; c Centre for Law, Justice and Society, De Montfort University, Leicester, UK; d Athens University of Business and Economics, Greece; e University of Macedonia, Greece; f University of Milan, Italy; g Radboud University, Nijmegen, the Netherlands; h Karolinska Institutet, Stockholm, Sweden; i Erasmus University Medical Centre, the Netherlands; j Research Centre Jülich, Institute of Neuroscience and Medicine (INM-1), Germany

**Keywords:** Neuroimages, Anonymization, Pseudonymisation, Neurodata, de-identification, MRI, Data protection

## Abstract

For a number of years, facial features removal techniques such as ‘defacing’, ‘skull stripping’ and ‘face masking/blurring’, were considered adequate privacy preserving tools to openly share brain images. Scientifically, these measures were already a compromise between data protection requirements and research impact of such data. Now, recent advances in machine learning and deep learning that indicate an increased possibility of re-identifiability from defaced neuroimages, have increased the tension between open science and data protection requirements. Researchers are left pondering how best to comply with the different jurisdictional requirements of anonymization, pseudonymisation or de-identification without compromising the scientific utility of neuroimages even further. In this paper, we present perspectives intended to clarify the meaning and scope of these concepts and highlight the privacy limitations of available pseudonymisation and de-identification techniques. We also discuss possible technical and organizational measures and safeguards that can facilitate sharing of pseudonymised neuroimages without causing further reductions to the utility of the data.

## 1 Introduction

Advances in imaging technology have led to significant changes in the nature, size and variety of neuroimages collected, processed, stored and shared. A range of largely open platforms are today available to facilitate international sharing of neuroimages. This growing movement of neuroscience towards open data sharing and the increasing collaborations between neuroscientists across geographic boundaries raises critical questions for the privacy and confidentiality of research subjects. The increasing prominence of data protection regulations, notably the European Union's General Data Protection Regulation (GDPR) and the United States' Health Insurance Portability and Accountability Act (HIPAA) (White et al., 2020) have increased attention to open data sharing initiatives and how data subject privacy issues are addressed.

Brain images that include facial regions can be used to recognise the research subjects (Schwarz et al., 2021). Conventionally, researchers are encouraged to apply techniques involving removal or blurring of facial features from the images before sharing to address privacy concerns. The underlying rationale is to achieve anonymity which allows for open sharing according to data protection laws (Nichols et al., 2017). Recent studies demonstrate, however, that these techniques not only reduce the utility of the data (de Sitter et al., 2020) but also are insufficient in achieving anonymity (Ravindra and Grama, 2019; Schwarz et al., 2019). This raises ethical, legal and scientific challenges for sharing of neuroimages. Therefore, this paper addresses the question: how can neuroimages be processed under available legal provisions to preserve data subjects’ privacy while retaining the scientific utility of the data? We intend to shed light on the tension between privacy and scientific utility.

This paper starts with descriptions of what we mean by *neuroimages* and provides a much needed conceptual clarity on the concepts of anonymization, pseudonymisation and de-identification of neuroimages. A clear understanding of these concepts is pertinent given their different jurisdictional origins, interpretations and implementations which raise challenges for often internationally trained researchers who are expected to comply with practical regulations. We then explore the common approaches/techniques to remove direct and or indirect identifiers in neuroimages, highlighting their impact on the scientific utility and their limitations in guaranteeing anonymity. We then identify measures and safeguards that can facilitate responsible processing of neuroimages in a way that will protect the privacy and confidentiality of data subjects without further emphasis on impairing the usefulness of the data used for analysis. This paper makes contributions to both research and practice: it improves legal and technical understanding of anonymization/pseudonymisation/de-identification of neuroimages and identifies legal positions on how neuroimages can be shared without overemphasizing the total removal of direct and indirect identifiers to preserve scientific utility. These insights are of interest to individual researchers, funding agencies, platform providers, local/(inter)national policy makers and members of institutional review boards.

## 2 Neuroimages

Neuroimaging refers to the use of various techniques (imaging technology) to create images of the structures, or function of the nervous system. These *in vivo* imaging techniques generate data that allow for a better understanding of the nature of healthy, as well as functionally impaired, human brains and the ‘‘underpinnings of mind and behaviour’’ (Gorgolewski et al., 2016a). Neuroimaging comprises many different data modalities (such as MRI, fMRI, diffusion MRI, CT, SPECT, PET, CT/CAT, Cranial ultrasound, and Functional ultrasound imaging). Note that within this paper, we focus on neuroimaging techniques which always display anatomical structures, besides some of them also being able to capture functional data. Techniques, such as magnetoencephalography (MEG), electroencephalography (EEG), intracranial electroencephalography (iEEG), or near-infrared spectroscopy (NIRS) that solely record the function of the brain and not its anatomy are therefore not considered here as neuroimaging techniques in the narrow sense. Nonetheless, the reader should keep in mind that functional brain data - just like behavioural and/or cognitive data - bears the risk of de-identification (see e.g., (Nishimoto et al., 2020). Otherwise known as brain imaging, neuroimaging has grown from its earliest conceptualisation as ‘neuroradiology’ (Fulham, 2004) to revolutionize the way the brain and its functions are understood in research and application (Poldrack, 2017). Images generated by neuroimaging techniques, particularly high resolution images of the anatomical characteristics or of the functional activation patterns, raise a number of concerns related to privacy and confidentiality of research participants. This is because of the availability of technologies that can generate recognizable images of the participant's facial features from them (Kulynych, 2002) including: 1) facial reconstruction and recognition technology, 2) technology that recognizes other patterns of indirect features that can be used for re-identification (such as 'brainprints'). Neuroimages can thus be described as personal data even when all direct information (e.g., the face) relating to the participant are removed. Such data are potentially identifiable given the presence of ‘brainprints’ which can be referred to as indirect identifiers and can lead to image-based re-identification. These concerns are further increased by efforts to share de-identified or pseudonymised neuroimages with ‘brainprints’ intact, for different research and non-clinical applications, including neuroprediction (Langenecker et al., 2018), neuroforecasting (Knutson and Genevsky, 2018), neuromarketing (McClure et al., 2004) and other consumer technologies. The potential re-identifiability of neuroimages presents ethical, legal and technical challenges for researchers, but also highlights the unique nature of such data.

### 2.1 Uniqueness of neuroimages

Article 4(1) of the EU General Data Protection Regulation (GDPR) specifies that ‘personal data’ means any information relating to an identified or identifiable natural person (‘data subject’); an identifiable natural person is one who can be identified, directly or indirectly.’ The European Court of Justice (ECJ) has confirmed that the *possibility* of identification is enough to consider some data personal (Borgesius, 2017).

In recent years, scientific studies have demonstrated that, like facial features and fingerprints, the brain anatomy is also highly individual and that it is possible to identify individuals based on specific neuroanatomical features (Gage and Muotri, 2012; Miranda-Dominguez et al., 2017; Valizadeh et al., 2018). These studies assert that the uniqueness of the brain anatomy is determined by the combination of genetic factors, individual life experiences, and stochastic processes (White et al., 2020). Neuroimages can be quantified to extract these individual brain differences and, for scientific reasons, relate them to external factors of interest. The brain has individualized structures and patterns that have been referred to as ‘brainprint’ (Aloui et al., 2018; Wachinger et al., 2014), ‘connectome fingerprint’ (Finn et al., 2015), ‘connectotype’ (Miranda-Dominguez et al., 2017). With the use of Linear Discriminant Analysis (LDA) and a modified version of the Weighted K-Nearest Neighbor (WKNN), Valizadeh et al. (2018) demonstrated that a combination of a relatively small number of neuroanatomical features is enough to identify individuals through neuroimages. Using scans from the Human Connectome Project, Byrge and Kennedy (2019) found that a small ‘‘thin slice’’ or random sample of the connectome was sufficient as a unique individual identifier.

This uniqueness underlines recent findings that individuals can be identified using neuroimages (Horien et al., 2018; Miranda-Dominguez et al., 2017; Schwarz et al., 2019; Waller et al., 2017). The implication of these findings is that neuroimages are inherently identifiable personal data and should be given the same privacy and confidentiality considerations as facial photographs (Schimke et al., 2011). They have also been characterized as novel forms of biometrics called ‘hidden biometrics’ which involve the use of specific medical or clinical data to identify individuals (Nait-Ali, 2011). In a study using MRI, Aloui et al. (2011) used brain characteristics as a biometric tool to identify individuals. This research was given credence by Chen et al. (2014) who designed a verification system for identity authentication based on the uniqueness of the brain. Their system produced results with a high degree of accuracy via pattern recognition. As an emerging biometric modality, Aloui et al. (2018) posited that the discriminative signature of the brain captured by neuroimages can provide a recognition rate of 99.6%. Neuroimages can thus be said to contain distinct structural and neural activity patterns that define each person and which may not be faked as fingerprints can (Geller et al., 1999). An individual's whole brain image is therefore quintessentially highly identifiable. That said, unlike a photo of the face, we are unable to observe or record the characteristic features of an individual's brain as they go about in their daily life. Thus, to be identified, individuals' brain imaging data must be either accessible in another database that links to their identity, or personally post their brain imaging data on social media, as some children have done after receiving a photo of their brain following a research based MRI (White et al., 2020).

As a hidden biometric, openly sharing neuroimages requires the consideration of the ethical principles of privacy and confidentiality. The EU General Data Protection Regulation (GDPR) (2018) amplifies this requirement and sets very high standards for ensuring the respect of data subjects’ rights to privacy and confidentiality. Considering the recent concerns about the potential exploitations that can be achieved by third parties from sharing of such personal data, e.g. as summarized by Rocher et al. (2019), the GDPR aims to ensure a stronger degree of protection. The GDPR, in essence, provides much greater control to participants regarding how they want their data to be used. These principles are at the heart of research ethics and researchers should offer the participants the opportunity to allow their data to be shared, and for those participants who agree with sharing, the researchs must assure that data protection and appropriate ethical/legal requirements are followed. Under the GDPR, protecting personal data involves use of technical safeguards that consist of the removal of all direct and or indirect identifiers in any personally identifiable data to enhance privacy and confidentiality. This is referred to as anonymization, pseudonymisation or de-identification.

### 2.2 Anonymization, pseudonymisation and de-identification

In describing the concept of **anonymization** as it was contained in the EU Directive 95/46/EC, Article 29 Working Party referred to anonymization as a type of data processing that involves the removal of sufficient elements from the data in such a way that a natural person can no longer be identified by using ‘‘all the means likely reasonably to be used’’. A similar definition can also be found in international technology privacy standards such as ISO 29100:2011, where it is defined as the “process by which personally identifiable information (PII) is irreversibly altered in such a way that a PII principal can no longer be identified directly or indirectly, either by the PII controller alone or in collaboration with any other party”. This is an irreversible process that should produce anonymous information which is defined in the GDPR as “information which does not relate to an identified or identifiable natural person or to personal data rendered anonymous in such a manner that the data subject is not or no longer identifiable”. It should be noted that the GDPR does not use the word ‘anonymization’ but focuses rather on the outcome of the process.

Most often in literature, anonymization is used synonymously with **de-identification** which is a concept used by the US Health Insurance Portability and Accountability Act (HIPPA) privacy rule for medical data (Freymann et al., 2012; Malin et al., 2011). HIPAA provides regulations describing when protected health information (PHI) can be used or disclosed for research purposes by covered entities (45 CFR § 164.501). One of the primary differences between the EU's GDPR and the US's HIPAA, is that whereas the GDPR is applicable to all personal data related to EU citizens and residents, the HIPAA applies only to the use of data within covered entities (45 C.F.R. § 160.103). Covered entities include insurance companies, health plans, health care providers, hospitals, and other institutions that relate to patient care. Covered entities are allowed to use and disclose PHI for research with the consent from the individual, or without individual consent under specific circumstances. These ‘specific circumstances’ are defined by 45 C.F.R. § 164.512(i) as the following: (a), obtaining a waiver by an institutional review board or equivalent medical ethics committee; (b), obtaining confirmation by researchers that they will use the data only to design a research protocol or for similar preparatory purposes necessary prior to implementing a research protocol. In addition, the researcher will not remove PHI from the covered entity and must agree that the data is necessary to make important decisions regarding the implementation of the study (i.e., risk/benefit ratio); or (c), when the use or disclosure is solely for research on the PHI of deceased persons, the PHI is necessary for the research, and, at the request of the covered entity, documentation of the death of the individuals about whom information is being sought 45 CFR 164.512(i)(1)(iii)); or (d) limited data sets coupled with a data transfer agreement [see 45 CFR § 164.514(e)]. The researcher ought to communicate that data will be used only for research on the PHI information of the deceased and the data is necessary for the study and documentation of the death of the individual.

Non-covered entities do not fall under HIPAA. Examples of non-covered entities that may collect PHI include devices that digitally capture information via wearables (e.g., heart rate, activity level, sleep, etc.) and upload this data to a database, recreational genetic databases (e.g., 23 and me), registries that are not housed within covered entities. Research conducted by non-covered entities fall under the regulations defined by the ‘Common Rule’. However, some institutions consist of covered and non-covered entities under the same roof. In these cases, the situation may arise that only the covered entity must comply with the HIPAA requirements under the Privacy Rule. PHI not held by a covered entity can be used and disclosed without regard to the Privacy Rule. However, specific state regulations such as the “Federal Policy for the Protection of Human Subjects” or the Common Rule still apply.

Section 164.514 of HIPAA provides two approaches (rule-based and probabilistic) to meet the required standards of de-identification that do not suggest irreversibility. The US law provides that a covered entity or what is referred to in the GDPR as the data controller may assign a unique code to de-identified data that can permit re-identification by the same entity (§ 164.514(c)). Unlike anonymization therefore, de-identification allows re-identification and this is the fundamental difference between anonymization and de-identification.

The robustness of the anonymization process on the other hand is dependent on consideration of “all” “likely” and “reasonable” means of re-identification of data subjects ‘‘either by the controller or by another person to identify the natural person directly or indirectly’’ (GDPR, Recital 26). This test of identifiability includes consideration of the cost, time and availability of technology required for re-identification (GDPR Recital 26). It is a test that should be made in accordance with suggestions made by the CJEU in *Breyer,* which clearly stated that information is personal data even if it requires legal and additional practical means to make a person ‘identifiable’ (Borgesius, 2017). This is a relevant test to ascertain what can be categorized as personal data and thus covered by the provisions of the GDPR. Therefore, when a dataset previously determined to be anonymized fails the test of ‘cost’, ‘time’ or ‘technology’, the data becomes personal data that has gone through a **pseudonymisation** process. In the GDPR, pseudonymisation is defined in article 4(5) as the processing of personal data in a way that it can no longer be attributed to a specific data subject without the use of additional information. One can therefore argue that any processing that allows the use of additional, direct or indirect identifiers/attributes, to re-identify the data subject falls under the category of pseudonymisation. That means, de-identification is closer in meaning to pseudonymisation than anonymization. Even a low risk of re-identification as permitted by de-identification specifications would disqualify data from being classified as “anonymized data” under the GDPR. Both pseudonymised and de-identified data still contain some risk of re-identifying the corresponding natural person. From this understanding, what is considered by US HIPAA as de-identified data and no longer falling under ‘protected health information’ (https://www.hipaajournal.com/de-identification-protected-health-information/) would be considered pseudonymised data which is still considered personal data according to the EU GDPR.

Taking the GDPR-based definitions for anonymization and pseudonymisation into account, and considering the ongoing technological progress in the field of machine learning, nowadays nearly all processes performed on raw and derived data that are associated with a specific natural person can at most only be classified as “pseudonymised”. This fact changes the historical view on “anonymization” of neuroimages, where the removal of direct personal information (facial structures, real names or contact information, etc) was sufficient to fully anonymize the corresponding data. Today, even if a neuroimaging data is de-identified, the remaining data still contain information that can be used for re-identification. For this reason, the anonymization of data requires more than the typical de-identification of direct or indirect personal data and includes more drastic measures such as cross-subject aggregation and randomization procedures. Unfortunately such real anonymization procedures typically have a high impact on the scientific value/potential of the data as will be discussed in the next section.

## 3 Pseudonymisation of neuroimages: scientific utility and open sharing

**Fig. 1:**
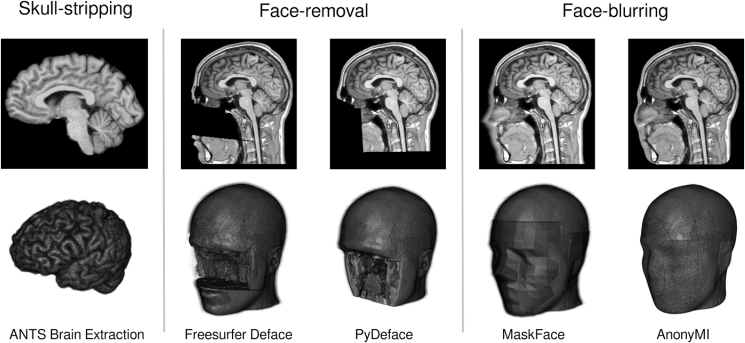
Examples of three of the four major groups of techniques to pseudonymize MRIs (cf. main text) (Omer Faruk Avants et al., 2011; Milchenko and Marcus, 2013; Gulban et al., 2019; Mikulan et al., In Press). Group 1: Skull-stripping example; Group 2: Face-removal examples; Group 3: Face-blurring examples. Group 4: Face-replacement (not shown in this figure). For all groups: top row displays sagittal views of pseudonymised MRIs; bottom row displays 3D representations of the same pseudonymised MRIs. All images were created from the MRI of a subject who provided informed consent.

The pseudonymisation of neuroimages and specifically MRIs constitutes a challenge as, to protect the subject's identity without limiting the scientific utility of the data, it should prevent re-identification whilst retaining as much information as possible. Several methodologies for achieving pseudonymisation exist which can be classified into four major groups which we will summarize focusing on their application to MRIs. The first group corresponds to skull-stripping techniques which extract the brain from the MRI volumes (see Fig. 1, left column), discarding everything else (for a review see (Kalavathi and Prasath, 2016). These methods preclude the possibility of using facial features, head geometries and subject-specific distinctive marks (scars, malformations, etc.) for identifying the subject, but are sensitive to several factors (e.g. population, MRI intensity, etc.) which make them relatively failure-prone (Fennema-Notestine et al., 2006). Another drawback is that they limit the possible analyses that can be performed on the data, especially for procedures that take advantage of head geometries (i.e. M/EEG source localization, sEEG coregistration, automatic segmentation, etc). The second group is formed by face-removal methods (see Fig. 1, middle column), which retain parts of the skull and the skin but partially or completely remove the facial area (Bischoff-Grethe et al., 2007; Schimke and Hale, 2011). These approaches are less error-prone and require less manual intervention than skull-stripping methods but still substantially modify the data due to the removal of the nose, eyes, cheeks and parts of the skull. The third group (see Fig. 1, right column) corresponds to face-blurring techniques, in which the whole head, skull and face geometries are retained but the face, ears and subject-specific marks are covered or blurred. For example, MaskFace (Milchenko and Marcus, 2013) works by identifying the voxels around the surface of the face and “flattening” them; and AnonyMI (Mikulan et al., In Press) by cropping the facial area (and any other area selected by the user) with a low-resolution 3D model of the head and filling the area between the skin and the inner skull with random numbers that follow the distribution of values of the subject's skull. These techniques offer the highest degree of data preservation but might carry a higher re-identification risk due the increased amount of information they conserve. The fourth group is formed by a recently developed technique in which the face of the subject is replaced with a new face (Schwarz et al., 2021). It works by identifying the facial area, replacing it with the face obtained with a facial template (obtained by averaging several faces) and finally normalizing the intensity values to match the original image. This method avoids the issues of face-removal techniques which is unique but still modifies the original geometry of the subject's head.

### 3.1 Impact on scientific utility of neuroimages

Neuroimaging datasets are information-rich resources whose uses can extend beyond the original publications that they accompany. For example, part of the data collected in the context of the Human Connectome Project (Glasser et al., 2016; Van Essen et al., 2013) have already been re-used in high impact publications (Deco et al., 2021). For this reason, the myriad of possible analyses makes it difficult to establish a pseudonymisation approach that, at the same time, is univocal and guarantees re-usability for each and every purpose. For example, a study focusing on the structural analysis of a specific brain structure and/or malformations could share an MRI dataset of skull-stripped brain images, because it doesn't need to consider bone, skin and soft tissue (Nalepa et al., 2020). On the other hand, when MRIs are shared as part of EEG studies, facial masking or removal techniques may induce geometrical distortions impeding accurate source modelling analyses that take advantage of head geometries (Hallez et al., 2007). In this respect, few studies have quantitatively tested the re-identification risk and the geometrical preservation offered by the state-of-the-art pseudonymisation methods (Budin et al., 2008; de Sitter et al., 2020; Mazura et al., 2012; Prior et al., 2008) and only one study evaluated these two aspects simultaneously (Mikulan et al., n.d.). Treating these two aspects simultaneously is of paramount importance to find a balance between utility in neuroimages and compliance with current legislation regarding data protection. In a study that evaluated how removal of facial features affect MRI, de Sitter et al. (2020) concluded that methods commonly used for the removal of facial features lead to failures of automated volumetric pipelines.

Another important aspect to discuss when considering the scientific utility of common pseudonymisation methods is how user-friendly they are. Indeed, the less friction experienced by researchers when applying a tool, the more likely it is to be properly used. In this respect, the ideal method should be fast and customizable; it should allow creating templates for particular datasets (possibly age specific); it should provide a user interface to mask specific areas of an individual subject that could lead to increased re-identification risk (e.g. scars); it should allow running analysis on multiple subjects automatically, making it easy to process large datasets, and have both a command-line and a graphical interface (essential for users with varying computer skills).

### 3.2 Privacy concerns

In addition to reducing the usefulness of these data, it has also been revealed in recent re-identification (de-anonymization) attack studies on such ‘anonymized neuroimages’, that the presumed anonymity provided by these techniques is compromised by both the uniqueness of the image and availability of advanced pattern recognition technologies (Ravindra and Grama, 2019; Schwarz et al., 2019). This means that datasets often referred to as ‘anonymized’ can mostly be reidentified (Knoppers et al., 2012; Loukides et al., 2010; Rocher et al., 2019; Rothstein, 2010). In their study, Rocher et al. (2019) concluded that it is possible to correctly re-identify 99.98% of Americans in any dataset using 15 demographic attributes and irrespective of applying anonymization techniques. This puts into question the appropriateness of openly sharing such data believed to be anonymized. The implication is that many of the available privacy protection approaches that inform open sharing of biomedical data do not qualify as ‘anonymization techniques’. This is especially true with large-scale data sets, with considerable heterogeneity of data. These studies highlight the challenges of achieving complete anonymity which can most times depend on availability of technology and expertise.

Using a novel CycleGan framework, Abramian and Eklund (2019) demonstrated that face blurring may not provide adequate protection against motivated attacks to de-anonymize de-identified neuroimages. Their experiment on a multi-site MRI dataset including T1, T2, PD, MRA and diffusion data from 581 subjects showed that it may be possible to reconstruct facial features from ‘anonymized’ images using generative adversarial networks (GANs), which is a technique that can be used to create new data from a training set through the competition between two neural networks. Schwarz et al. (2019) have also demonstrated the possibility of re-identifying research participants from de-identified MRI scans with the help of face-recognition software. After de-facing with popular and available techniques, Schwarz et al. (2021) discovered that face recognition was able to re-identify 28%–38% of research participants. Furthermore, Venkatesh et al. (2020) demonstrated that a subject's functional connectivity matrix that is statistically derived from resting state fMRI data of the Human Connectome Project (HCP) can still be used to re-identify the subject with an accuracy of 95% using geodesic distance.

These findings make one wonder what the privacy implications of a malicious attack on publicly available neuroimages will be considering that advanced deep learning technology can lead to facial recognition of participants from neuroimages (Parks and Monson, 2017; Prior et al., 2008). A de-anonymization attack conducted by Ravindra and Grama (2019) which relied on novel techniques of analysis, revealed the theoretical possibility of not only re-identifying the individual subjects, but also the tasks they were performing during the scan and potentially other corresponding patient data like progression of disease, behavioral traits, sex, gender and contact details. This is of great concern to research infrastructures facilitating the sharing of multi-dimensional neuroimages especially with regard to balancing the necessity to share neurodata and the need to ensure privacy and confidentiality.

### 3.3 Open sharing predicated on ‘anonymization’

The increasing tendency to openly share neuroimaging data (Poldrack and Gorgolewski, 2014, Poldrack and Gorgolewski, 2017) is based on the assumption that techniques that obscure facial features guarantee anonymity of the data subjects (Nichols et al., 2017). As open neuroscience and collaborative research continue to gain traction within and across institutions and countries, large-scale research infrastructures, tools and platforms are facilitating the practice of open sharing of neuroimages and their associated metadata. A number of international and large-scale neuroimaging databases are currently sharing pseudonymised (or de-identified) neuroimages based upon this assumption. These developments are driven by research funders’ aim to provide open access to data, the need of the scientific community to make best use of data as a crucial resource, and importantly, to offer greater opportunities to address important scientific questions that will benefit society. Centralized and mostly open-access neuroimaging repositories/archives are being used to overcome significant technical and organizational challenges associated with data sharing and transfer. These developments are further required by the demands of scientific rigor, reproducibility and the opportunity to reduce cost, given that there are considerable logistic and monetary expenses associated with collecting and processing of neuroimages (Eickhoff et al., 2016; Poline et al., 2012), and are the driving force behind new requirements for sharing by funding agencies. Furthermore, advancements in data and metadata standardization schemes, such as BIDS (Gorgolewski et al., 2016a) have facilitated the necessary management and harmonization effort and increased sharing, access and reuse of neuroimaging data (Gorgolewski et al., 2017) in accordance with the FAIR (Findable, Accessible, Interoperable, and Re-useable) data principles (Wilkinson et al., 2016).

Processing and sharing human neuroimages within and across borders comes with the demand to consider additional regulations pertaining to privacy and confidentiality owing to the uniqueness of the datasets. As we have identified in section 2, all data that originate from a single person are potentially identifiable and this can also include derived data (e.g. intra-subject statistics) as well (Venkatesh et al., 2020). Sharing such data constitutes a form of processing that falls within the scope of data protection laws and often involves a variety of stakeholders and legal jurisdictions.

Pseudonymisation or de-identification provides the grounds for open sharing for the majority of neuroscience databases. For example, OpenNeuro; 1 makes it clear that only de-identified data are shared, and data providers have to explicitly agree that datasets shared do not contain any identifiable personal health information as defined by HIPPA and are not subject to GDPR provisions. De-identification or Pseudonymisation can be argued to give a false sense of security that allows open sharing without considerations for technical and organizational measures that can provide additional security and privacy of the data. Other repositories openly sharing pseudonymised individual subject neuroimages include: Brain-development.org,^2^ Brain/MINDS; 3 and the international neuroimaging data-sharing initiative (INDI).^4^ In the case of Brain-development.org, datasets include tabular files with date of birth and other personal information of participants (see Table 1). Many project specific, institutional and nationally funded databases also allow redistribution of shared pseudonymised datasets. This open sharing practice is based on previous understanding of the impact of available techniques on neuroimages and may not be an intentional breach of privacy. It is important, therefore, for researchers and platform providers to be aware of not only the scientific impact of these techniques and tools, but also their privacy limitations which should inspire alternative and responsible ways of sharing.

**Table 1:** How some neuroscience databases currently implement technical and organisational measures.^a^.

Name of database	Data protection by design and by default				DUA^b^
	Pseudonymisation	Access control			
		Account registration with strong authentication process	Simple registration with no/weak authentication	Unclear/inconsistent access control	
EBRAINS	✔	✔			✔

OpenNeuro	✔		✔		

ADNI	✔	✔			✔

DABI	✔			✔^c^	✔

Brain-Development.org d	✔				

BrainMaps	✔				

Brain/MINDS	✔		✔		

Caltech Subcortical Atlas	✔				

International Neuroimaging Data-sharing Initiative	✔			✔^e^	

Open Access Series of Imaging Studies (OASIS)	✔	✔			✔

SchizConnect	✔		✔		✔

The Donders Repository	✔	✔			✔

Human Connectome Project (HCP)	✔	✔			✔

a How informed consent and encryption are applied in these databases was not reviewed for this paper because this will entail contacting individual data providers who originally collected the data from patients. Additionally, application of encryption at rest or on transit is not obvious from information on the websites.

b DUA here does not include licence agreements that do not touch on privacy issues.

c Access control solely managed by data providers but the process is unclear.

d Also provides a spreadsheet with date of birth and other details of all participants.

e Users need to log in on NITRC, but some of the data is also simply available from http://fcon_1000.projects.nitrc.org/indi and on Amazon S3 as well with no access control.

## 4 Responsible sharing of neuroimages

Having discussed the nature of neuroimages and the problem of anonymization, pseudonymisation and de-identification as well as their impact on the law and practice of data sharing, this section explores what needs to be done, in order to ensure that sharing of neuroimages is done responsibly, i.e. that it is legally compliant, socially acceptable as well as scientifically valuable. Both HIPAA and the GDPR provide legal guidance on the processing of health data. However, the focus of this section is the GDPR considered by some (Raab, 2020) as an example of a data protection law with both ethical and legal dimensions. Jwa and Poldrack (2021) focused on how the US regulatory landscape shapes sharing of neuroimages. However, there is a question to be asked of whether HIPAA is robust enough to address the emerging privacy concerns related to neuroimages, but that is a question for another research or paper.

### 4.1 Neuroimages as health, sensitive and special category data

The GDPR introduces a definition of health data as ‘‘personal data concerning health … of a data subject which reveals information relating to the past, current or future physical or mental health status of the data subject’’ (GDPR, 2018, Recital 35; Article 4(15)). Most importantly, they include ‘‘information derived from the testing or examination of a body part or bodily substance … and any information on, for example, a disease, disability, disease risk, medical history, clinical treatment or the physiological or biomedical state of the data subject’’. It doesn't matter who, where or what device is used to collect the data. As data that can reveal past, current and future health of a natural person, neuroimaging data qualifies as health data and consequently a special category of personal data or sensitive data according to Article 9 of the GDPR. Although this definition brings some level of clarity as to the identification of certain types of research data as health data, it does not completely remove the uncertainties of how to process health data especially for individual researchers considering varied interpretations of existing exemptions.

The understanding is that the processing of health data poses greater risk to the fundamental rights and freedoms of natural persons and therefore merits higher protection than other types of personal data. Indeed, processing of such data is prohibited unless one of ten listed lawful bases in Article 9 of the GDPR applies (such as explicit consent; employment; vital interests; made public by data subject; carried out by a not for profit organization; legal claims; preventive or occupational medicine; public interests in the area of public health; archiving purposes in public interest, scientific or historical research or statistical purposes; substantial public interest on the basis of union or state law). The complexity of identifying this lawful basis, the need to respect the purpose limitation principle, prohibitions on data transfers to third countries and the imperative to adopt stronger security measures for health data combine to create confusion over how to share health data (Eiss, 2020). In the following section we will discuss what possibilities are available for legitimate processing of neuroimaging data.

### 4.2 Technical and organisational measures and safeguards for processing neuroimages

So far, this paper has pointed out the difficulty of rendering neuroimages completely anonymous and the inadequacies of available techniques in providing a full guarantee of anonymity following accurate re-identification via facial reconstruction and recognition, as well as other pattern recognition techniques (e.g., machine learning). Although it may not be wholly accurate to state that neuroimages can never be anonymized, it should never be assumed that neuroimages are anonymous and predicating open sharing on available approaches without any form of risk assessment is therefore not legally adequate (White et al., 2020). Determination of what qualifies as anonymous data should be on a case-by-case basis. In addition, these approaches degrade the utility of the data and emphasis on further removal of features from the images may adversely affect the outcome of analyses. The responsible scientific goal should therefore be to identify how to process these images in a way that the rights of research participants are respected while scientific discovery is furthered. The first step in identifying relevant safeguards and measures for lawfully processing brain images is the acknowledgment of the inherent privacy risks and the limitations of available privacy preserving techniques. Data sharing platforms are becoming aware of this and are developing additional measures to legitimize sharing.

**Fig. 2:**
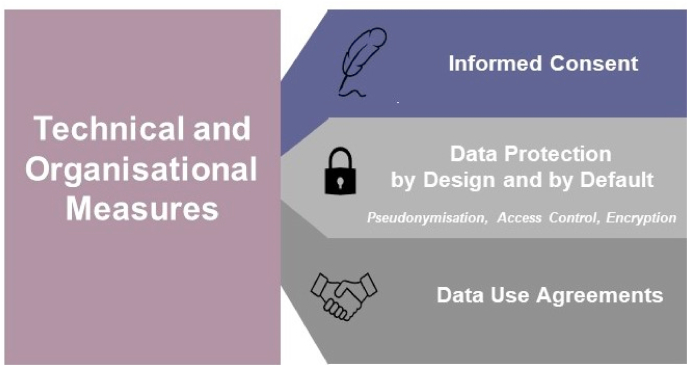
Technical and Organizational measures and safeguards for processing neuroimages.

At the core of legitimate data processing of data are basic lawful bases detailed in the GDPR article 6 (1)(consent, performance of a contract, compliance with a legal obligation, protection of vital interests, in the public interest, legitimate interests) and at least one condition listed in article 9(2) when processing special categories of data. In addition to these, sharing of personal data (brain images) shall be subject to appropriate safeguards – organizational and technical measures-so as to protect the rights of the data subject and respect key principles of minimisation (article 89 GDPR). The following section will be dedicated to discussing some of these key legal bases for processing and legitimate measures which are shown in Fig. 2.

#### 4.2.1 Informed consent

For most research data generated under a study protocol, an important lawful basis for data processing under article 6 is consent, which is also one of the exemptions for processing of health data under article 9. This is distinct from informed consent which is a core prerequisite for enrolling research participants for any biomedical study as embedded in both the *Oviedo Convention* and the *Declaration of Helsinki*. Typically, obtained informed consent from research participants limits the use of data to a specific research project or does not indicate commitment to share or publish data beyond the project team (Spence et al., 2018). Where data sharing is acknowledged, researchers often guarantee that only ‘anonymized’ data will be shared which excludes pseudonymised data as explained in section 2. Obtaining informed consent for a research study may not meet the requirements of explicit consent required for lawful processing of personal data. Researchers need to be aware that while consent for processing can be integrated into the informed consent for research, the form ought to be designed in a way that meets the consent requirements necessary for sharing. The data subjects must clearly understand that their pseudonymised data may be shared within the international research community and the researchers' role in mitigating the risk of re-identification through the consent form. Data subjects must be given the choice to explicitly consent to a specific processing (including sharing) of their brain images (pseudonymised or not). The Open Brain Consent (OBC) project (Open Brain Consent working group, 2021) provides neuroimaging researchers with template consent forms that explicitly consider privacy and the GDPR. But because ‘it is often not possible to fully identify the purpose of personal data processing for scientific research purposes at the time of data collection’, Recital 33 allows ‘consent to certain areas of scientific research when in keeping with recognised ethical standards for scientific research’. This is often interpreted as broad consent for scientific research which has been revealed to have flaws when applied to processing of sensitive data (Holm and Ploug, 2019). To avoid the complex issue of broad consent in health data, the OBC template makes it clear that data may be used for future research projects, including research in the field of medical and cognitive neuroscience.

Consent is not the only – and may not always be the appropriate-legal basis or exemption for processing health data for scientific research purposes. Alternatively, lawful basis for processing may for instance be public interest (Article 6(1)(e)) or the legitimate interest of the data controller (Article 6(1)(f)), combined processing in the interest of public health (Article 9(2)(i)) or necessary for scientific research purposes (Article 9(2)(j)) which are conditions for processing special category data. But when processing is based on another lawful basis in article 6 other than consent and one of the exemptions in article 9 (2) GDPR, the ‘ethical’ requirement of informed consent for participation in biomedical research will still have to be met (EDPS, 2020).

A number of reasons exist that can affect reliance on consent as the lawful basis for processing neuroimaging data, including member state laws as allowed in Article 9(4) which has consequences on how to respect data subjects' rights. For instance, UK guidance on health research states that consent should not be the appropriate lawful basis for processing data for health and social care research. Opinion 3/2019 of the European Data Protection Board (EDPB)^5^ has further observed that consent is not an appropriate lawful basis for processing in research activities where there is a clear imbalance of power between the data controller and the data subject. An example of such a situation is when obtaining consent may be difficult or impossible due to the health conditions of the data subject. In neuroimaging research, therefore, a robust evaluation of the specific research circumstances needs to be carried out to determine if consent is appropriate. When relying on consent as a lawful basis for processing brain images as health data, the EDPB has made it clear that Recital 33 does not take precedence over the conditions set out in articles 4(11), 6(1)(a), 7 and 9(2)(a) of the GDPR. Consent given for certain areas of scientific research still requires well-described purpose and greater transparency. A careful assessment of data subjects’ rights, the sensitivity of the data, the nature and purpose of the research and the considerations of relevant ethical standards are required (Ibid). In addition to the identification of adequate lawful basis for processing, article 89(1) of the GDPR also requires additional safeguards to be established for personal data processed for scientific research purposes.

#### 4.2.2 Data protection by design and by default

The data protection protocols, ‘‘privacy by default’’ and ‘privacy by design’ can also provide legitimate safeguards that allow the sharing of brain images. These are legal requirements by the GDPR that imply data protection through technology design; integrating necessary safeguards into the processing stream that can protect data subjects' rights (see article 25 GDPR). They include the implementation of technical measures that can address privacy issues, bordering on collection, processing, accessibility and storage, by default. As GDPR pointed out, these measures should be ‘‘designed to implement data protection principles, such as data minimisation, in an effective manner and to integrate the safeguards … to meet the requirements’’ of the regulation (see article 25(1)). Important to this is the consideration of the cost of implementation, nature, type, scope, circumstances, purpose and potential risks of the data processing which is best done via a Data Protection impact assessment (DPIA). The GDPR places the responsibility of identifying the exact protective measures required in the hands of the data controllers which often are individual researchers. Persons or entities (organisations) as controllers will need to remain accountable in respect of their processing activities; what data are collected, how they are processed and stored and who gets access to them. One way to identify and implement these measures is by conducting a Data Protection Impact Assessment (DPIA). A DPIA is particularly necessary where processing involves the use of new technologies, in this case, imaging technologies and is likely to result in high risks to the rights and freedoms of data subjects (see article 35, GDPR). The DPIA is therefore used to assess data processing workflows, identifying privacy risks and developing approaches to mitigate these risks. For brain images that are sensitive health data, several protective measures are required to satisfy the GDPR requirements of ‘privacy by design’ and ‘privacy by default’ in addition to cybersecurity. These include: pseudonymisation, encryption and access control.

1. **Pseudonymisation**: Article 89(1) GDPR particularly singled out pseudonymisation and where possible anonymization as part of the appropriate technical safeguards for the data processing ‘‘for archiving purposes in the public interest, scientific or historical research purposes or statistical purposes’’. As the possibility of fully anonymizing brain images is up for debate, pseudonymisation becomes a viable option that can serve two purposes; preserve significant utility of the data and ensure respect for the principle of data minimisation thereby contributing to data protection by design.
2. **Encryption:** In addition to pseudonymisation, encryption was mentioned severally in the GDPR (see articles 32; 6 Par. (4e), recital 83) as an appropriate technical and organizational measure to ensure the security of processing. The sensitive nature of brain images means that sharing over networks requires additional security to prevent data modification and reduce the risks when transferring the data to authorized entities. Encryption and decryption approaches such as Data Encryption Standard (DES), Advanced Encryption Standard (AES), the Hash Function and encryption based on chaotic systems that can be applied to medical images have been identified in literature (Guo et al., 2016; Preishuber et al., 2018; Yun-Peng et al., 2009). In addition, an approach (encryption and decryption network DLEDNet) that leverages deep learning techniques for image-to-image translation and image denoising has also been proposed (Ding et al., 2021). On transit and at rest, encryption approaches that meet current standards contribute to meeting GDPR privacy requirements and reduce the liability of a data controller in cases of breaches. For some data sharing e-infrastructures where encryption on transit may be challenging, infrastructure level encryption may suffice if pseudonymisation and technical access control are implemented. Indeed, the loss of encrypted data storage platforms is not considered a data breach as per article 83(2) (c) of the GDPR.
3. **Access control:** Article 25(2) makes it clear that privacy by design measures shall ensure that by default, personal data are not made accessible to an indefinite number of natural persons or to bad actors. One way of achieving this is via technical access control mechanisms. Brain images, like genomic data, ought to be shared through controlled access models that involve authentication or authorization. Access to individually identifiable, albeit pseudonymous, neuroimages can be granted by project-specific or infrastructure-level data access review committees (ARC). The controlled-access platforms use ARCs to vet the researchers requesting access and ensure that the data are used only for identifiable legitimate purposes that align with a lawful basis. ARCs are no panacea and their use may introduce a conflict of interest that mirrors the overall tension this paper describes. ARCs are typically instituted by the organisations running the data processing infrastructure. They therefore have a fundamental interest in broadening their reach making data available, so they are not neutral arbiters of the public good. They therefore should be seen as one component of the mix of organisational and technical measures. At this point there is little empirical evidence to assess whether such conflicts of interest do arise in practice and how severe they are. If they are shown to be problematic, then other solutions might need to be considered, such as independent third-party ARCs.

#### 4.2.3 Data use agreements

Besides the aforementioned strategy to implement privacy-by-design, another organizational and legal measure to consider is the use of data use agreements (DUAs) or data use terms. In large neuroimaging projects and consortia it is common that data is shared under an explicit agreement with the data user that imposes restrictions regarding potential re-identification (Jwa and Poldrack, 2021). For example in the Human Connectome Project data is organized at different levels of sensitivity; the least sensitive data (the neuroimages) are shared under a relatively liberal agreement which forbids attempts to re-identify or contact participants. The second level of data that is more sensitive and that includes the family structure and twin relations, is shared under a DUA that is more restrictive: it also forbids publishing examples of neuroimages of individual participants in papers, in case that might disclose information about the family structure and participant identifiers to readers of the paper that have themselves not agreed to the data use terms. Other large consortia and projects in which neuroimages are collected and shared, such as ABCD, UK BioBank, and ABIDE also implement restrictions on the reuse of data by means of data use agreements.

Most neuroimaging studies are, however, executed by individual researchers working in smaller labs. Upon publication of the research findings and trying to also make the research data available under FAIR data principles, these researchers often face the limitation that they do not have the legal expertise, nor support, to set up data use agreements. Academic institutions such as universities are understandably hesitant in individual employees engaging in legal contracts with external parties; as a consequence DUAs need to be agreed upon not only by the researcher and her/his PI, but also by the research director, legal department and eventually the board of the institution.

A notable exception for institutional DUAs has been implemented by the Donders Institute at the Radboud University, who make data, including neuroimages, available through the Donders Repository. In collaboration with the RU legal department and security officer, a DUA was created that explicitly deals with potentially identifiable neuroimages, and imposes restrictions on re-identification and linkage attacks. Individual researchers at the Donders Institute who want to share the data for their study can do so through the Donders Repository using this RU-DI-HD data use agreement. The DUA imposes restrictions on re-identifying participants and on redistribution of data, and requires people that wish to reuse the data to be identified. Another reason why a DUA is important is the necessity to incorporate relevant GDPR specificities of EU member states.

### 4.3 How neuroscience databases apply these measures/safeguards

In order to understand how de-identified or pseudonymised neuroimages are shared globally, we reviewed some neuroscience databases. The following were our criteria for inclusion of databases: a. platforms sharing living human neuroimages particularly MRI and fMRI b. sharing individual subject's data c. open for external users to download data. Applying these criteria ensured that databases with similar elements were reviewed. Neuroscience databases that share only aggregated data or animal data were not included in this review. Private repositories hosted by authors only for the purposes of publication were also excluded. The process of identifying these databases started with internet searches using the search terms: *neuroscience databases sharing neuroimages* and *platforms sharing neuroimages.* The initial search revealed 51 platforms sharing neuroimages of which only 31 are sharing human neuroimages. This was subsequently reduced to 13 databases sharing data from individual subjects as shown in Table 1. Information from the websites of these databases including access policies and procedures were then read in detail to understand specific platform approaches to sharing. We did not aim and do not claim, in any form, that this list is exhaustive. We are only interested in demonstrating how high-profile neuroscience databases are applying the above technical and organizational measures for sharing neuroimages as of August 3, 2021. The aim here was to provide an overview of what measures and safeguards available in some global repositories/databases.

Each of the above measures (pictured in Fig. 2) on its own is not sufficient justification for open sharing of brain images. A combination of all these measures is necessary to provide adequate levels of privacy for identifiable neuroimages. A number of databases have established at least one of these measures such as Donders,^6^ EBRAINS,^7^ ADNI; 8 and OASIS.^9^ Some of these databases have levels of access control that are not sufficient to guarantee security. Among these include OpenNeuro, Brain/MINDS and SchizConnect where a simple registration without adequate confirmation of the user's ID provides access to data. None has, however, implemented all the above measures (informed consent, data protection by design and by default and DUA). But it is important to note that effective implementation of these measures is often hindered by the absence of internationally standardized and understandable framework for consent, DUAs, access control and pseudonymisation for neuroimaging researchers. This is partly because of the different regulatory requirements and conditions but most often due to the varied interpretations of these requirements. Individual researchers, institutions and data archives/repositories require clear ideas of what conditions/requirements are supposed to be met for data collection, curation and storage that affects sharing of neuroimages.

### 4.4 Proposed computational solutions

Beyond the above legal mechanisms for sharing neuroimages, there have also been computational solutions proposed for information sharing, some of them also summarized in White et al. (2020). These methodologies do not share raw image data, but aggregated data of previously collected or published information, or as result of distributed (remotely executed) computations. In any case, to coin such techniques and frameworks as privacy-preserving, formal privacy guarantees have to be provided which we will discuss briefly in the following paragraphs.

The de-facto mechanism for providing formal privacy guarantees is Differential Privacy (Dwork, 2006), which bounds the probability of identifying the presence of a particular record in an aggregate dataset through noise utilization. Nevertheless, the amount of noise to be used should be carefully calibrated so that the utility is maintained in a dataset, while the privacy guarantees are also acceptable. This, however, is not a trivial task, as induced noise may significantly undermine data quality, leading to false results (Scheibner et al., 2021). Additionally, enforcing Differential Privacy may further complicate data publishing, as a sufficient amount of data should be curated and integrated to form a substantial corpus upon which noise may be applied.

Approaches discussed below may not have been initially designed to preserve privacy, but they share information through sharing computation results rather than raw data. In their majority, however, they do not provide formal privacy guarantees, which is a later advancement. In Li et al. (2020), federated analysis of neuroimages is performed using differential privacy. However, further research is expected, due to imposed noise/utility incurred trade-offs.

To begin with, there are methodologies based on sharing statistical information for specific areas of the brain and, in some cases, the correlation structure between these. Such an approach can be based on sharing peak coordinates extracted from published tables in previous studies (Fox and Lancaster, 2002). They propose an indexing system for mapping brain data in a multidimensional feature space. As this approach was proposed relatively early in the development of neuroimaging, no particular measures had been considered to enhance privacy, other than relying on aggregate statistics from other sources. Similarly, unthresholded statistical maps associated with scholarly articles are being shared by Neurovault (Gorgolewski et al., 2016b). Brain network maps are created in (Muetzel et al., 2016) which may allow for connectivity analyses. However, in these cases privacy is not strictly guaranteed since, as reported by White et al. (2020), even group averages can reveal unanticipated information about the individual.

Other proposed privacy-centric computational approaches include the Datashield (Gaye et al., 2014), ViPAR (Carter et al., 2016) and ENIGMA (Thompson et al., 2014). Datashield uses computation over summaries and does not quantify privacy or provide any guarantees against re-identification (Sarwate et al., 2014). ViPAR temporarily pools subsets of the data via encrypted transfer to a trusted server on which the automated analysis takes place (Carter et al., 2016). However, as indicated in Plis et al. (2016), this approach is bandwidth-demanding and does not address privacy issues deriving from direct data transfers, even when connections are encrypted. In ENIGMA, summary statistics over locally computed data are shared. ENIGMA performs meta and mega analyses on manually curated data, which requires both training and resources. This method for privacy preservation is mainly based on the fact that summaries are shared in cases of meta-analyses. However, as discussed earlier, this does not offer formal privacy guarantees.

One recent computational method is described in Baker et al. (2019) where authors propose decentralized joint independent component analysis (djICA) for fMRI images, in order to address privacy issues by offering decentralized computation, without the need to transfer data between data-holding parties. However, formal privacy guarantees are not provided in this case either. While the authors claim their methods to be easily extendable to accommodate formal privacy mechanisms, such as differential privacy, no empirical evidence is provided toward this direction. As differential privacy utilizes noise injection, assessing the impact of such a mechanism is necessary as data quality may be reduced.

It is evident that there is a tendency toward performing decentralized computations in order to preserve privacy. COINSTAC (Plis et al., 2016) moves toward performing federated analysis, in terms of stochastic gradient descent and in specific sparse regression, and parametric statistical testing. However, there is no indication of formal privacy guarantees employed in COINSTAC, although it is stated that this framework is suitable for its application.

It is evident that most of the aforementioned methods lack formal privacy guarantees considered suitable under GDPR guidelines. Furthermore, computation-based approaches exhibit additional drawbacks, as these techniques are mostly based on summary statistics over a number of raw data items. To perform these types of remote computations, they need to be predefined, implemented in appropriate scripts, shared and installed. This prevents establishment of hypotheses through data observation. Computational approaches also need to consider how to respect data subjects’ rights as mentioned in section 4.2.

Finally, it is important to mention that in order to deploy and maintain such analysis frameworks, centralized or decentralized Research Data Management (RDM) systems are deployed. The implementation of a decentralized RDM is beneficial to commonly shared data that have to be stored in a decentralized manner (e.g., because of institutional or ethical policies; cf. Hanke et al., 2021). Available remotely executable analysis frameworks (e.g., OpenMined^10^ or MIP (Redolfi et al., 2020)) often assume that a matching RDM on the data storage side is already in place. Moreover, for matching the remotely executable analysis framework with the used RDM, tools with built-in support for data versioning with metadata extraction and metadata-based search, such as DataLad (Halchenko et al., 2021), have to be implemented (cf. Hanke et al., 2021). Nevertheless, an RDM system does not typically take care of privacy guarantees in general, or the implementation of remotely executable analysis frameworks with proven privacy guarantees. Both aspects have to be deployed and maintained on top. In addition, setting up and maintaining a RDM system takes time and effort. To conclude, RDM systems play the role of a building block for data sharing and remotely executable analysis frameworks. Yet, additional measures and resources are required for achieving privacy preservation.

## 5 Conclusion

This paper touches on a pressing problem that many neuroscience researchers face when processing neuroimages, namely the question whether and how they can share these neuroimaging data. It is motivated by the recognition that there is a potential trade-off between openness that would promote the interests of science and data protection in the interest of data subjects. In order to shed light on how this trade-off is to be conceptualised and addressed, we asked the research question: how can neuroimages be processed under available legal provisions to preserve data subjects’ privacy while retaining the scientific utility of the data?

The discussion of technical measures that have traditionally been used to protect the identity of data subjects and their interpretation in the light of current legislation has shown that what used to be accepted as sufficient levels of protection is unlikely to be enough. The formerly predominant assumption that defacing and similar techniques make it practically impossible to identify the data subject has been disproven, rendering these approaches insufficient in light of recent legislation, notably the GDPR.

Looking at the technical and legal discussion in this paper one can thus conclude that the currently dominant reliance on technical measures to ensure data subjects’ privacy is scientifically dubious and therefore unlikely to be legally compliant. Publicly sharing data that contains enough information about the brain to be scientifically interesting is likely to lead to and facilitate data protection breaches. If this conclusion is correct, then it raises significant challenges to the neuroimaging community. The prevalent practice of making neurodata publicly available is no longer tenable and will need to be reconsidered. This may come as a surprise to some in the neuroimaging community but probably not as a surprise to many, as the topic is already widely discussed. The unrestricted sharing of neuroimaging data of individuals who are subject to the GDPR may well be illegal and already published data may have to be withdrawn from unrestricted public access.

The good news is that this does not mean that sharing of neuroimaging data for scientific purposes is impossible. Instead, it implies that the way in which neuroimages are shared will need to be reconsidered. We have discussed several mechanisms that will allow neuroimage sharing, calling for a suitable legal basis, data protection by design and default and other organisational measures such as the implementation of data use agreements. While making use of these measures will require important changes to the way in which the neuroinformatics community works, these changes are already visible in many data sharing infrastructures and can be made to work relatively easily.

While these changes are technically feasible, they will require a change of culture of the neuroimaging community. Culture change is rarely easy and quick, but in this case the law seems to have been faster than technology development which will require a quick response from neuroimage researchers. It is important to see that this requires responses at all levels of the research ecosystem. Neuroscience education will need to highlight these issues to new entrants to the field and established researchers and PIs will need to be trained in them. It is crucial, however, to ensure that individual researchers are not left alone to deal with them, as they will mostly lack the requisite legal knowledge and practical means to address them successfully. This means that there is an important role for research organisations such as universities to provide support structures, for research funders to require attention to data sharing in grant applications and for research policy makers to promote suitable ways of dealing with these questions. A crucial role will be played by the research infrastructures that are used for the sharing of neuroimages, who will need to agree on shared practices and standards.

Change is rarely welcome and one can expect resistance to such a fundamental change that is even accompanied by the threat of legal sanctions. We believe, however, that the neuroimaging community should see the analysis developed in this paper as an opportunity and embrace this change as a way of improving the way neuroimages are shared. It holds the opportunity to improve the quality of data and the scope for the scientific community to make use of it. Ensuring that neuroimaging data can be shared in a way that retains its scientific value and safeguards the privacy of data subjects thus opens the possibility of increasing the societal value derived from the data, encouraging the participation of data subjects. This, in turn, can increase and protect the overall societal acceptability and esteem that neuroscience as one of the most visible and crucial scientific disciplines enjoys.

## Funding

This work was supported by the 10.13039/100010661European Union's Horizon 2020 Framework Programme for Research and Innovation under the Specific Grant Agreements No. 720270 (HBP SGA1), No. 785907 (HBP SGA2) and No. 945539 (HBP SGA3).

## Declarations of interest

None.

## CRediT author statement

Eke, Damian: Conceptualisation, writing – original draft/review and editing Aasebø, Ida E.J: Conceptualisation, writing – review and editing Akintoye, Simisola: Conceptualisation, writing – review and editing Knight, William: Conceptualisation, writing – review and editing Karakasidis, Alexandros: Conceptualisation, writing – original draft Mikulan, Ezequiel: Conceptualisation, writing – original draft Ochang, Paschal: writing – review and editing Ogoh, George: Conceptualisation, writing – review and editing Oostenveld, Robert: Conceptualisation, writing – review and editing Pigorini, Andrea: Conceptualisation, writing- original draft: Stahl, Bernd Carsten: Conceptualisation, writing – original draft/review and editing White, Tonya: writing-original draft Zehl, Lyuba: Conceptualisation, writing – original draft/review and editing.

## Declaration of competing interest

The authors declare that they have no known competing financial interests or personal relationships that could have appeared to influence the work reported in this paper.

## Footnotes

1 See https://openneuro.org/.

2 http://brain-development.org/.

3 https://dataportal.brainminds.jp/.

4 http://fcon_1000.projects.nitrc.org/.

5 https://edpb.europa.eu/our-work-tools/our-documents/opinion-art-70/opinion-32019-concerning-questions-and-answers_en.

6 https://data.donders.ru.nl/.

7 https://ebrains.eu/.

8 http://adni.loni.usc.edu/.

9 https://www.oasis-brains.org/.

10 https://www.openmined.org/.
